# Implementing clinical guidelines to promote integration of mental health services in primary health care: a qualitative study of a systems policy intervention in Uganda

**DOI:** 10.1186/s13033-019-0304-9

**Published:** 2019-07-17

**Authors:** Edith K. Wakida, Celestino Obua, Seggane Musisi, Godfrey Z. Rukundo, Peter Ssebutinde, Zohray M. Talib, Dickens Akena, Elialilia S. Okello

**Affiliations:** 1 Department of Psychiatry, Mbarara University of Science and Technology, P. O. Box 1410, Mbarara, Uganda; 2 Department of Pharmacology and Therapeutics and Vice Chancellor, Mbarara University of Science and Technology, Mbarara, Uganda; 3 Department of Psychiatry, Makerere University College of Health Sciences, Kampala, Uganda; 4 Department of Psychiatry, Mbarara University of Science and Technology, Mbarara, Uganda; 5 Mbarara District Local Goverment, Directorate of Health Services, Mbarara, Uganda; 6 Department of Medical Education, California University of Science and Medicine, San Bernardino, USA; 7 Mbarara University of Science and Technology, Mbarara, Uganda; 8 Mwanza Intervention Trials Unit, Mwanza, Tanzania

**Keywords:** Uganda Clinical Guidelines, Uptake, Integration of mental health services, Primary health care providers

## Abstract

**Background:**

Clinical practice guidelines (CPG) are developed based on a synthesis of evidence regarding the best options for the assessment, diagnosis and treatment of diseases and are recognized as essential quality improvement tools. However, despite growing availability of CPG, research evaluating their use for mental disorders in Uganda is lacking. For a successful implementation of CPG to be achieved, a number of considerations need to be put in place.

**Objective:**

This study aimed to assess the feasibility and acceptability of the educational intervention that we developed towards improvement of the primary health care providers (PHCPs) uptake of the Uganda Clinical Guidelines (UCG) in integrating mental health services into PHC in Mbarara district, southwestern Uganda.

**Methods:**

This was a descriptive cross-sectional qualitative study with a semi-structured in-depth interview guide. The educational intervention we were assessing had four components: (i) summarized UCG on common mental disorders; (ii) modified Health Management Information System (HMIS) registers to include mental health; (iii) clinician’s checklist outlining the steps to be followed; and iv) support supervision/training.

**Results:**

Six themes emerged from the study while the components of the intervention formed the apriori subthemes. Key results based on the subthemes show: (i) summarized UCG: the participants liked the packaging stating that it eased their work, was time saving and user friendly; (ii) modified register: participants appreciated the modifications made to the register updating the existing record in the Health Management Information System (HMIS) registers to include mental health disorders; (iii) TRAINING and support supervision: the PHCPs attributed the success in using the summarized UCG to the training they received, and they further expressed the need to regularize the training in assessment for mental health and support by the mental health specialists.

**Conclusion:**

Our study demonstrates that the use of summarized UCG, modified HMIS registers to include mental health, training and support supervision by mental health specialists in implementing the UCG in integrating mental health at PHC settings is feasible and acceptable by the PHCPs in Mbarara district, southwestern Uganda. Given the need for improved mental health care in Uganda, this intervention could be rigorously evaluated for effectiveness, scalability and generalizability.

**Electronic supplementary material:**

The online version of this article (10.1186/s13033-019-0304-9) contains supplementary material, which is available to authorized users.

## Background

Clinical practice guidelines (CPG) are developed based on a synthesis of evidence regarding the best options for the assessment, diagnosis and treatment. They are recognized as essential quality improvement tools [1–3]. CPG are meant to be a one-stop-shop for implementers by providing synthesised information from systematic reviews regarding best practices [3, 4]. However, despite growing availability of CPG, research evaluating uptake has shown varied results [2, 4–7] and is indeed lacking in Uganda when considering mental health disorders. There is thus a need to understand the barriers to CPG implementation and use [8, 9] especially when it comes to mental disorders. Various studies indicate that multiple barriers prevent the successful adoption of, and adherence to CPG. These barriers include, but are not limited to the (a) lack of awareness of or disagreement with some content of CPG, (b) insufficient motivation to change, (c) inappropriateness of some CPG to local settings; and (d) time constraints due to high volume of patients and shortage of health care workers [10–12].

For a successful implementation of CPG to be achieved, a number of considerations need to be put in place. These include considerations such as detailed knowledge about the local context in which the guidelines will be implemented, active mechanisms that enforce the implementation/adaptation of the CPG, and local expertise to identify challenges and report them accordingly [11]. Systematic reviews suggest potential implementation strategies, such as audit and feedback, outreach and opinion leaders [13]. Additionally, evidence suggests that tailored, multi-layered approaches may do better than single-focused interventions [2, 14, 15]. In resource constrained sub-Saharan Africa (SSA), little has been done to ensure successful implementation of CPG that are meant to improve outcomes for individuals with mental illnesses.

Recent reviews determined that multilayered implementation strategies including educational materials or meetings along with reminders, and coordination by a member of the healthcare team were most likely to improve adherence to following CPG implementation [2, 14, 15]. The multilayered approaches to a successful implementation of CPG includes the use of individuals with in-depth knowledge to guide health care workers [16]; consideration of the opinions of local opinion leaders [13]; and the use of the checklists [17]. The use of outreach programs has been shown to strengthen a range of intervention components and approaches, as well as addressing the challenges in implementation [17, 18]. Furthermore, educational and dissemination interventions have been shown to lead to better uptake of the policy than interventions that target organizational change [19].

Despite the availability of evidence indicating possible remedies that can address the challenges of implementing CPG, a number of barriers do exist especially in low resourced SSA [20]. The barriers to implementation of CPG could potentially impair the effectiveness of an intervention to improve professional practice. Identification of these barriers forms the first step in designing policy strategies that can help address them [20]. In the case of the Uganda Clinical Guidelines (UCG), its usage was found to be impractical due to unavailability, the bulk of the book, and the lack of cues to use the guidelines [11, 21].

**Fig. 1:**
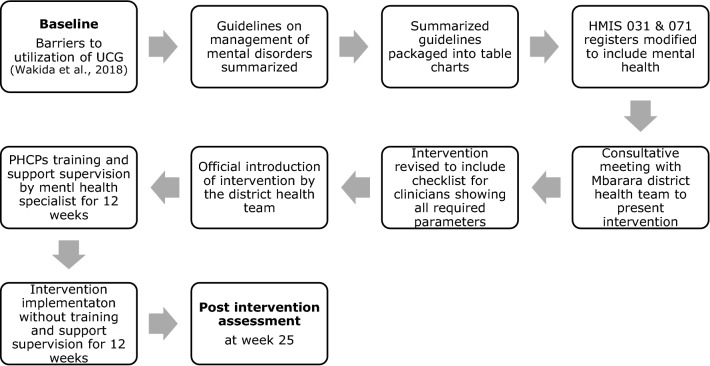
Sequential steps in developing and implementing the educational intervention

Based on the results of our initial study that identified perceived barriers and facilitators to PHCPs ability to integrate mental health services into PHC in Mbarara, Uganda [11], we developed a policy educational intervention. The intervention had four components: i) summarized UCG on common mental disorders; ii) modified Health Management Information System (HMIS) registers to include mental health; iii) clinician’s checklist outlining the steps to be followed; and iv) support supervision/training (described in the methods section) to promote usage of the UCG in integrating mental health services into PHC (Fig. 1).

### Aim of the study

This study aimed to assess the feasibility and acceptability of an educational intervention towards improvement of the PHCPs uptake of the UCG in integrating mental health services into PHC in Mbarara district, southwestern Uganda. It was hoped that this would help close the void in assessing and reporting on mental health disorders from PHC settings in Uganda.

## Method

### Study design

This was a descriptive cross-sectional qualitative study that was assessing the feasibility and acceptability of an educational intervention from the perspective of the PHCPs in rural Mbarara district, Uganda. We used a semi-structured interview guide with probing and open-ended questions that allowed the respondents to bring in unique examples and detailed descriptions.

This approach is methodologically similar but contextually different from our previous publications on barriers to integration of mental health services into PHC, and health systems constraints in rural Mbarara district of Uganda [11, 21]. The study was designed by the primary author, EW in consultation with the other authors ESO, ZT, PS, SM, GR and CO.

### Study setting

The study was conducted in Mbarara district approximately 270 km by road, southwest of the capital city, Kampala. Mbarara is the administrative capital of southwestern Uganda and it boarders Ibanda and Kiruhura Districts to the north, Kiruhura and Isingiro Districts to the east, Isingiro and Ntungamo Districts to the south, and Sheema District to the west. Demographically, Mbarara district lies between coordinates 00 36S, 30 36E and covers an area of 1846.4 km^2^ with a population of 472,625 of which 242,547 (51.3%) are females [22].

Mental health services in Uganda are provided at health center (HC) III (sub-county) level, with subsequent referrals to HC IV (county level), district hospitals, regional referral hospitals and national referral hospitals [23, 24]. Each health facility level (except HC III) is expected to have general doctors (medical officers), clinical officers (Diploma level Medical Assistants), nurses and midwives, and psychiatric nurses. The HC IIIs do not have general doctors but have all the other cadres of service providers. Mbarara district has thirteen HC IIIs and four HC IV and provision of health services is spearheaded by the district health department responsible for curative and preventive healthcare [25]. The HCs that were included in this study are located in rural Mbarara district.

### Description of the intervention

We based our study on the results of our initial study that identified perceived barriers and facilitators to PHCPs ability to integrate mental health services into PHC in Mbarara, Uganda [11]. We then developed an educational intervention for PHCPs to improve on their usage of the UCG in mental health assessments and reporting of the disorders in their PHC settings. The intervention had four components including: (a) summarized guidelines of common mental disorders as provided in the UCG [24]; (b) modified Health Management Information System (HMIS) registers (031-outpatients department, and 071-antenatal) to include columns of selected mental health disorders; (c) clinician’s checklist outlining the steps to be followed in a PHC setting in Uganda; and (d) support supervision and training (Additional file 1). We used the human centered approach [26] of working with the PHCPs, as end users, to come up with a solution to the perceived barriers so that they can better function at their highest possible level in implementing the integration policy (see Fig. 1).

The Intervention: This had four components shown below.

- Summarized guidelines: Content about management and treatment of mental disorders was summarized from the UCG [24]. The selection of the mental health disorders was informed by the disorders listed on the HMIS data collection report form used by the Ministry of Health [27]. Three categories of mental disorders were selected as follows: (1) Mental (depression, and bipolar), (2) Neurological (epilepsy), and (3) Substance use disorders (Alcohol abuse). Each disorder was illustrated in terms of treatment and management, indicating what is to be done at which facility level (Additional file 2). The information was packaged in form of an easy to use table chart.
- Modified register: Four extra columns were added in the existing HMIS registers 031 (Out-patients department, OPD) and 071 (antenatal); each labeled with one of depression, bipolar disorder, epilepsy and alcohol use disorders. The modification was done for the purpose of this study and with permission from the office of the district health officer to try its feasibility and acceptability by the PHCPs. The registers are paper based so there was need to supply sufficient copies for the intervention period which lasted 6 months (24 weeks).
- Clinician’s checklist: A checklist for clinicians was developed for the PHCPs to follow when they receive patients in the consultation room. It was a computer-generated illustration of all the processes including mental health assessment (Additional file 3). This item was suggested by the District Health Officer (DHO) of Mbarara district (opinion leader) in the spirit of promoting holistic integration of services at PHC. The checklist was packaged in form of a wall chart and was pinned in the clinicians’ rooms as prompts.
- Support supervision and training: A mental health specialist from Mbarara Regional Referral Hospital where the PHCPs refer all the cases they cannot manage at their levels was engaged during the intervention. The specialist provided training based on the disorders summarized in the guidelines, including depression, bipolar disorder, epilepsy and alcohol use disorders. This training included coaching on which treatment (medicines and the dosage), and management (counselling, referral or to give a follow up date) as provided for in the UCG. In addition, mentorship and real time support was provided to the PHCPs answering challenging scenarios faced with a patient, as well as auditing the modified registers for correctness and providing feedback where there were mistakes.

### Study duration

The intervention lasted 6 months (November 2018 to April 2019) making a total of 24 weeks which were divided into 12 weeks for intervention with all the components listed above, and 12 weeks with no training and support supervision but with access to the registers, checklists, and summarized guidelines. The intervention was officially introduced by the District Health Officer, DHO of Mbarara district and his team at two intervention sites in Mbarara district—one Health Center (HC) III, and one HC IV. The HMIS 031 and 071 registers were officially retracted for a period of 24 weeks to pave way for the intervention study. The DHO’s team acknowledged the study as very relevant, timely, and had the potential of addressing the challenge of integrating mental health services into PHC in the Uganda.

Follow up interviews were conducted at the end of April 2019 (in week 25) to assess the feasibility and acceptability of the intervention. Although all PHCPs at the two intervention sites took part in the intervention, only those who had taken part in the study identifying barriers and possible solutions were eligible to take part in the post intervention assessment. This paper only describes what was done at post intervention.

### Study costs

We calculated the actual direct costs of the intervention per site as detailed in Table 1. There were other costs related to implementation of the intervention like fuel costs to the study sites. The actual value was not directly allocable to the intervention because it served more than transportation to the HCs as each time a trip was made to the facilities. Training was done on site, so no venue hire costs were incurred.

**Table 1:** Summary of actual direct cost of the intervention

Item	Quantity	Unit cost UGX	Total UGX	Total USD^a^
Summarized guidelines	10 copies*2 sites	20,000	400,000	110
Register	3 copies*2 sites*6 months	40,000	1,440,000	263
Clinician’s checklist	10 copies*2 sites	10,000	200,000	55
Training/supervision	1 person*5 days*12 weeks	100,000	6,000,000	1644
Total cost			8,040,000	2202

^a^Exchange rate USD: UGX = 3650. Noteworthy from the direct costs involved is the fact that some of the components perceived to have worked well such as the summarized UCG were relatively cheap

### Study participant’s and recruitment

Participants who took part in the study included a clinical officer, nurses and a midwife from two HCs (III and IV) in Mbarara district; only those who participated in our initial study [11], directly assessed patients, were willing to participate in all study related procedures, and provided a written informed consent were eligible to be included in the post intervention assessment. One HC III and one HC IV were selected using simple random sampling out of the four HCs (two HC III and two HC IV) that were part of the initial study.

### Data collection and analysis

A semi-structured in-depth interview guide was used to collect data from PHCPs at the two intervention sites. The interviews were conducted in English, the official national language, and backed by field notes. In-depth interviews were conducted by the lead author (EW) together with a trained research assistant (MN) in the last week of April 2019. All interviews were conducted in person at the respective HCs, and all participants provided voluntary written informed consent. Each interview lasted approximately 60–90 min, was audio recorded, and transcribed verbatim by the research assistant. The transcripts were checked by EW against the audio recordings for correctness of information and securely stored; they can only be accessed by EW, ESO and CO.

Data were thematically analyzed [28, 29] by ESO and EW with the help of a qualitative software Atlas.ti version 8 [30]. Codes and categories for understanding PHCPs perceptions on the feasibility and acceptability of the intervention were independently constructed from the data by ESO and EW; and through consensus, a codebook was developed. The initial coding was done by EW after discussion with CO a senior researcher, ZT a health policy expert, and GZR a mental health specialist. To address reflexivity, the first author (EW) conducted the initial interviews together with a trained research assistant to ensure consistency in the conduct of the interviews. The rest of the interviews were conducted by the trained research assistant (MN). EW conducted the initial analysis of the data, shared and discussed the emerging themes with the rest of the authors.

### Governance and quality control

#### Study oversight

There was an oversight team during the entire intervention period to ensure that the study was being implemented as planned. The team comprised of ESO a qualitative methods expert and medical anthropologist, ZT a health policy expert; CO a senior researcher; PEA research ethics representative; PS the District Health Officer; and AD, SM and GZR as mental health experts.

#### Data quality procedures

These were observed to monitor the performance of the intervention as follows: (a) the Mbarara district health office was involved in the development of the final intervention materials; (b) registration sheets were provided for the mental health specialist, the lead researcher/author (EW), and the PHCPs to sign at the end of each support supervision; (c) attendance sheets were signed by participants at the end of each training session, (d) involvement of a mental health specialist in training and support supervision/mentorship, (e) preparing standard training materials, so as to avoid differences in information given to PCHPs at the different HCs.

## Results

We had anticipated interviewing all the participants (eight in total from the intervention sites) who took part in the initial study as well as the intervention. However, at the time of follow up, we found that one participant at HC IV level had been transferred to another HC midway the study period. Therefore, only seven in-depth interviews were conducted, with three participants from HC IV and four from HC III. Table 2 shows a summary of the participants’ characteristics.

**Table 2:** Summary of participants’ characteristics

	Age	Gender	Health cadre	Level of education
1	48	F	Midwife	Certificate Midwifery
2	33	F	Nursing Officer	Diploma Nursing
3	32	F	Enrolled Nurse	Certificate Nursing
4	33	F	Psychiatric Nurse	Certificate in Mental Health Nursing
5	50	F	Senior Nursing Officer	Diploma Nursing, Diploma Midwife
6	36	F	Nursing Officer	Diploma Nursing
7	39	F	Clinical Officer	Diploma Clinical Medicine

All the participants were female aged between 31 and 50 years at the time of the study. Segregated by position or health provider category, there was one clinical officer, one senior nursing officer, two nursing officers, one enrolled nurse, one midwife, and one psychiatric nurse, in the health facilities. Views of all the participants were included in the analysis and contribute to the conclusions in our study.

Six themes emerged from the study while the components of the intervention formed the apriori subthemes. These were: (a) relevance of the intervention, (b) impact of the intervention, (c) format of the intervention, (d) personal reactions to the intervention (e) adherence to guidelines, and (f) perceived increased work load and time constraints.

### Relevance of the intervention

In this study, relevance was looked at in terms of suitability of the intervention to the local setting. Below is how the participants responded when asked about how appropriate the different elements of the intervention were. These included the summarized guidelines, the additions to the register and the training provided:

#### Summarized guidelines

Participants liked the packaging of the summarized clinical guidelines on mental health conditions and noted that they were more user-friendly and attractive to use than the big volume book of the UCG for the management of all common conditions. One participant at HC III level stated:

*“I tell you the chart (summarized guidelines) is good, it has content about management and treatment of mental illnesses…and it is not a very big volume we would be avoiding, so we do not feel we should dodge it; we feel that a big volume (referring to the Uganda Clinical Guidelines book) is for professors but that is not there with the summarized guidelines”* ***(Nurse 6, site 1)***

The participants appreciated the relevance of the summarized guidelines because they were able to quickly know which treatment to offer to the client. They decried inaccessibility of the UCG because of limited copies supplied and personalization of the few available copies. A nurse participant said:

*“Actually, they are very relevant because before, we never had those table charts (with summarized guidelines), I could assess a patient but couldn’t tell which first line drug to give; and the Uganda Clinical Guidelines book was not accessible because the copies were limited and personalized so you find they are personalized and taken away from clinical rooms… but those table charts (summarized guidelines) have not been taken by anyone, since they are always there on the table… So, they are very easy and important to use”* ***(Nurse 4, site 2)***

#### Modified register

In the HMIS registers, unlike other conditions such as tuberculosis and malaria, there is no provision for collecting data on mental health. In this study, we modified the register to include columns for capturing data on mental health. Participants were glad that all data could now be collected from one location as one expressed below:

*“The register is okay, because that part of the mental was now included in the same register, there is no movement that you are looking for it, it is there and you just fill in the same register”* ***(Nurse 6, site 1)***

Previously, most participants did not consider alcohol use disorders as a mental health problem and were not tracking them. With the modified register, they were now able to document the number of cases they get. One participant stated:

*“… we had not been tracking alcohol addicts; at least now we have that part of alcohol use disorders… that part at least now it works; we know the number of patients in a month who abuse alcohol”* ***(Nurse 4, site 2)***

#### Training and support supervision

Participants expressed that the training would be most effective if regularized and repeated so that at any one time there were trained people given the many transfers of staff which happen. A participant stated:

*“It was acceptable, and we wish it could be regular because at certain times staff may be transferred, they bring new ones, so at least the new ones also need to come on board… and when they get it from the mouth of the supervisor, it works better, it needs may be to be regular”* ***(Nurse 3, site 2)***

The training on mental disorders was perceived to be very timely and useful given that the participants had not received such training before as illustrated in the quote below:

*… the mental disorders were being neglected so the training came at a perfect time when we really needed someone to come up and talk about the mental problems. So, it was really suitable (* ***Nurse 2, site 2)***

### Impact of the intervention

#### Summarized guidelines

Reflecting on how they had been working, the participants felt that the presence of the summarized guidelines on the mental conditions significantly eased their work and that this was very time saving as it included all the steps that a clinician needed to follow in terms of treatment and management. The feeling is illustrated in the quote below:

*“…my work was simplified especially after talking to the patient and you look through the table chart (summarized guidelines) and just know where the treatment is and what to give…. Before, I would assess the patient and make a diagnosis but then the treatment becomes a problem, so I would have to consult the doctor on which first line drug to give, but this process was time consuming… but since the summarized guideline became available, I consult them and it ends there* ***(Nurse 4, site 2)***

#### Modified register

We found that inclusion of columns on mental health in the register acted as a cue for the PHCPs to assess for mental conditions. The participants indicated that initially they used not to pay attention to assessment of mental conditions but now they have to as illustrated below:

*“It is helping us to assess more of our clients for mental conditions, at first with the other register (HMIS), even if you would see a client and do not assess for mental health, there was no problem; but now when you see a client and it reaches a time to leave the clinic when you have not assessed for mental, when you reach there and you say ummm… I really need to assess for this”* ***(Nurse 5, site 1)***

Participants who worked in the HIV clinic, however, reported a challenge of having nowhere to record information about the mental status of their clients and yet they acknowledged that they were identifying them. It is important to note here that the modified and provided registers were HMIS 031 for OPD and HMIS 071 for antenatal records only. If there were any other registers at the HCs, they were not modified. The participants added that patients who attended the HIV clinic did not go to the OPD so there was a missed opportunity of recording and attending to the mental conditions of these HIV patients. Besides, the prepared HIV reports did not provide for documenting information on mental health status of the patients with HIV. This dilemma was expressed by one of the participants:

*“the only challenge we have in my department is that HIV clients with mental conditions are not captured in the HIV register… we identify and diagnose in their files; they don’t normally go to the OPD register where their mental health could be captured; so, I don’t have anywhere to register them… So, when we are making the HIV report, there is no mental health reporting, they are not reported anywhere. I don’t know how best way to do it, whether to transfer them (patients in the HIV clinic with mental conditions) to OPD because I don’t register them anywhere and yet they need to be managed for their mental conditions as well. I should get where to register those clients (with mental conditions) in the HIV clinic. I don’t know but I am not sure the best way out because the ART clients don’t go to out*- *patients department”* ***(Nurse 2, site 2)***

#### Training and support supervision

Most participants reported experiencing valuable support from the mental health specialist during the training and supervision which included knowledge sharing, giving feedback on how to use the intervention materials, and what to do in the areas that needed improvement as expressed below:

*“It has been there, they are supportive, because even doctor (the mental health specialist) when he used to come for supervision and you have your questions, he would give you time and you ask him. He taught us on the conditions that we ask him, we share the challenges together and he is able to give us feedback”* ***(Nurse 5, site 1)***

### Format of the intervention

#### Summarized guidelines

In comparison to the book format of the clinical guidelines provided at the health facilities in Uganda, all the participants indicated that the summarized format in the study was more user friendly. Below is a verbatim quotation of one participant:

*“it has been helpful …before I used to refer every mental condition because I have a weakness of reading much, but now I can read through this summary and see how I should manage the conditions that would have had to be referred to other (higher) levels…”* ***(Midwife, site 1)***

#### Modified register

Concerning the modified registers, all the participants acknowledged that the format was user friendly. Although more columns were added, participants observed that they were not so many to bother them. They suggested that the addition should be maintained. They should not go back to the old official register which did not have provision for mental health conditions. One participant nurse expressed herself thus:

*“that format is good, I have nothing to say about changing it, but if the Ministry of Health doesn’t want to continue with this book (referring to the modified register), at least they could get one special for me to be recording those patients with mental conditions in their own register rather than bringing back the other former register (official register)”* ***(Nurse 4, site 2)***

#### Training and support supervision

In regards to the format of training and support supervision, the participants thought that this should be provided more regularly with structured sessions to allow for both theory and practical sessions. One respondent said:

*“Support supervision should be done quarterly, and for the trainings to have at least three sessions a day. If the training is to run for a week, maybe in a day we could have three sessions to allow people to understand, and then the other time we go for practical’s or scenarios from participants during that time”* ***(Nurse 6, site 1)***

### Personal reactions to the interventions

#### Summarized guidelines

Most of the participants attributed their success during the intervention to the presence of the summarized guidelines on common mental disorders and the training. They noted that initially they did not know what to do when faced with a client having a mental disorder. Indeed they had developed negative attitudes towards them. But with training on how to use the summarized guidelines for assessing for mental conditions, the participants reported a positive attitude as illustrated below:

*“… actually before, we could just refer even it was not necessary, and because when you have a knowledge gap you are not comfortable seeing this client because you think you may get a challenge or mismanage the client so we could have a negative attitude on clients with mental illnesses, but now we find it easy to see clients with mental illnesses”* ***(Nurse 5, site 1)***

*“I think this intervention has opened our eyes to think more on mental health and to know what to do, whether to refer or to give drugs…it has really helped us. We are managing more patients now than before the policy intervention” (* ***Nurse 3, site 2)***

Most participants indicated that they did not like the idea of the clinician’s checklist pinned in examination rooms to remind them on what to do. With training, they felt that they now knew all the procedures and the mental conditions that came to them. They instead felt that such effort should be directed to getting the clinicians to bring integration of mental health care to the level of the other medical conditions, as expressed below:

*“… The health workers these days, they have that (pointing to the clinician’s checklist) here (pointing to the head)…they will do it first thing even if you woke them up from bed, they will do this for you (following the clinician’s checklist). What you need to do is to help this one (pointing to mental health) to become like this (TB and malaria) at the finger tips of the clinicians”* ***(Clinical Officer, site 1)***

#### Modified register

Participants appreciated that the additions to the register were not put in a separate document. They appreciated that the mental conditions were added to be among the conditions they usually see and record in the HMIS registers. The participants did not find a problem assessing for mental health disorders. They felt that they could handle the mental conditions when faced with a client as stated below:

*“I think it is okay because first of all this register (referring to the intervention register) has all the parts of the HMIS register that we see as our usual conditions; it is not a separate register, the conditions are all in one register…and when assessing clients, we usually assess them for many things so I do not think that would be an issue assessing for mental illnesses… it is rather helping us to make right diagnosis”* ***(Nurse 5, site 1)***

#### Training and support supervision

Some participants expected and wanted more than what the intervention was able to offer. These were happy with the knowledge they gained during training and support supervision. However, they also expected that the intervention would be able to support full time presence of a mental health specialist at the health facility to continue with the supervision. They said:

*“We expected to learn more and more as we have gained this knowledge… we thought the mental health specialist who provided training and support supervision would stay and work with us, and also moves with us to the mental health clinic where he works”* ***(Clinical Officer, site 1)***

To some the intervention exceeded their expectations. They felt that the intervention was able to equip them with knowledge and skills to identify, manage or refer mentally ill patients. These said:

*“We were hoping to be more conversant with mental health…I think no mental health client will escape from us because after knowing all this, you can identify and know how to handle them…those who we cannot handle we will refer. Our expectations were to know how to handle mental cases and how to be conversant with them…and we have achieved it”* ***(Nurse 3, site 2)***

Some participants preferred the training to be structured differently with some flexibility that allowed the health workers to participate in the training while others attended to the patients.

*“what I heard people complain about was the time for training during working hours, so, but if say this week, this group is to train then the other week, this other group also trains, so that the work remains moving at the health unit and the other group remains moving not coming like in working hours then you rush with work so that we sit and train. So, you find people are restless they worry about neglecting clients, they are not concentrating, they are even few like so”* ***(Nurse 4, site 2)***

*“Only that the duration of the training period was short…we would enjoy and learn more things if trained for one week not at the facility; especially us midwives we would be on and off, we couldn’t settle. I prefer one week off site and we learn all the conditions at once. But on site you can leave people on this condition you go to conduct a delivery, next day you find they are on another condition”* ***(Midwife, site 1)***

### Adherence to guidelines

#### Over-enthusiasm

We found that participants at HC III level had assumed the role of treating and managing mental conditions they would have otherwise referred to HC IV. The reason they gave was that they had received training and felt more confident to handle any condition. In addition, they felt that they had the summarized guidelines, and psychotropic medicines at their disposal. They thus wondered why they should refer to another level. Below is a quotation from a nursing officer:

*“To me, why should I refer this patient when I was trained and I have the knowledge and skills, and the drug is here? You may find that the patient you are referring has no money for transport and even has no one to take care of that patient at higher levels… you want me to just give a referral letter and they go back home with their issues? To me, I think I would manage the patient and as I said I keep monitoring”* ***(Nurse 6, site 1)***

We probed further to understand why the participants at HC III no longer referred patients to HC IV as stipulated in the clinical guidelines. We found that some participants who had been in practice for a long time believed that they had the experience to handle the mental conditions they received. This was in addition to an attitude change about clients with mental disorders as reported below:

*“…of course, we have been in the practice for some time; when I say I have not referred, then I managed. But something that is severe and needs an admission, definitely that one I will not handle…and before, we would not even touch them (patients with mental illness) unless they were referred here for a refill. We would just send them to the parish for that visit when mental health conditions were being managed at the dispensary once a month, and when they would go there, we stopped”* ***(Clinical Officer, site 1)***

Failure to refer patients, however, led to another challenge at the HCs. Participants reported drug stock outs which they attributed to their perceived ability to identify and manage all patients with mental conditions. Initially, they used to refer everybody they suspected of having a mental disorder but now, they started treating serious conditions which they were not supposed to manage at their level of training. This resulted in drug stock outs of particular psychotropic medicines due to their limited supply. One participant said:

*“…we normally run out of stock of carbamazepine and amitriptyline, they are not there; and of course, they bring one tin each for the two medications, and then we have a lot of phenytoin and chlorpromazine. We do not know how to choose each; for example, carbamazepine or phenytoin”* ***(Nurse 2, site 2)***

When further probed about the issue of drug stock outs, the participants attributed it to over prescription of some medicines than others because of the perception that the conditions they treated required these particular medications. Some were more common than the others. One clinical officer stated:

*“…the first explanation is that we never order for them (psychotropic medicines), they just push them to us.…and secondly, I think health workers prescribe those medicines (carbamazepine and amitriptyline) very often. It could be the reason they are out of stock often; because for example chlorpromazine…there are very few patients who are on chlorpromazine, even those who come for refill, they are very few. Why?”* ***(Clinical Officer, site 1)***

### Perceived increased work load and time constraints

We asked the participants what they thought about the health workers time spent on assessing patients for mental conditions. They seemed surprised about the question. Most participants indicated that a clinician should not mind about the time spent on a patient with mental conditions because they spend time seeing patients. They argued thus:

*…but a health workers’ time to me I find there is no problem, we are supposed to know these things only that we didn’t have the knowledge, but since we are able to screen for TB, we are able to screen for malnutrition, we are able to screen for HIV, we can also integrate mental health and screen for it* ***(Nurse 5, site 1)***

On the other hand, however, some participants reported increased workload and limited time to fully attend to the patients. They felt that this had led to delegation of duties especially with documentation, thus resulting in gathering inaccurate data. They stated:

*“Now, we have a problem, the one who fills in the register and the one making clinical notes, those are two different people…when you see patients and at the same time fill the register, you take a lot of time on one person…so in order to save time, the health workers tend to push the work of registration to a different person… I think the nurses just don’t like registering because there is a lot of pressure, high workload due to many patients. That one has led us to receiving wrong data because of the people writing in the register… it’s a challenge I have seen, and I think we have to improve”* ***(Clinical Officer, site 1)***

#### Support supervision

The participants thought that support supervision for mental health should be handled the way other medical conditions like HIV, TB and malaria were prioritized. They did not think cost or time to provide supervision for mental health services should be an issue. The issue was to consider promoting and even identifying a focal person at district level to promote mental health activities. They said:

*“….the cost, I think the way they do support supervision for other conditions like HIV, they could also do for mental health because I think mental health has been put aside too much and I don’t know why; but I think with government since it is minding much on malaria, and gives out mosquito nets to people, and gives out HIV drugs to HIV patients, let it also do something for mental health… the way they have HIV focal persons at the district level, the way they have HIV focal persons everywhere, let it also be made for mental health”* ***(Nurse 4, site 2)***

The participants provided the following suggestions to the policy makers as a way of optimizing uptake of the integrating mental health services into PHC. First and foremost, the PHCPs who took part in the study believed that adapting the summarized version of UCG, and modifying the HMIS registers to include mental health would go a long way in supporting uptake of the policy. They felt that it was important to make this communication to the responsible persons as expressed in the verbatim quotation below:

*“To me what I would recommend, that at least you first sit with these people at the district level maybe if you have a say there, to continue with the modified register the way you made it (with provision for mental conditions), and to continue providing more table charts (summarized guidelines) the way you brought them. All the components used were good, so, I think you tell them that…”* ***(Nurse 4, site 2)***

#### District focal mental health person

The participants highlighted the need to have a mental health focal person at the district level who can help push the mental health agenda at the district level. This suggestion was made in light of the fact that other medical conditions were well catered for and data pertaining to the conditions was being collected. They said:

*“we get a focal person for mental health, because every Monday we send a message for malaria, for INH, for family planning, PMTCT, and also for TB…make an SMS showing how many mental conditions were recognized this week, how many people were diagnosed with any mental condition, you can break it down to different conditions, but if you don’t want, you can leave it like that…how many were treated…that is how we have been able to capture them”* ***(Clinical Officer, site 1)***

The participants also suggested the use of educational charts with illustrations of various mental disorders pinned in the HCs to help raising awareness among patients and their care takers about mental health. They recommended that the charts should provide basic information on how to recognise symptoms of mental illness and the steps to take.

*“We need charts, the big charts with illustrations of mental health conditions…you see like this flow chart of malaria, we can also have these flow charts for mental health; you can draw some conditions on the charts so that the patients can see and remember the pictures so that when they see someone like that in the community, they can accept them…when they get an attack, they can do ABC. That one can be very good compared to this (the clinician’s checklist)”* ***(Clinical Officer, site 1)***

## Discussion

This study showed that developing the education intervention on the mental health policy based on the human centred approach [26] provided opportunity for the for the participants to function better at their highest level. In addition, the study allowed us to understand its feasibility and acceptability from the perspective of the PHCPs. Studies have shown that efforts to achieve successful uptake of clinical practice guidelines need to fit in the local context [11]. Interventions tailored to anticipated barriers are likely to improve professional practice and, they need to be relevant to the end-user [20]. Getting the PHCPs to use the summarized UCG was the ultimate purpose of this study. Pantoja T et al. in their Cochrane Database of systematic reviews concluded that printed educational materials improve clinical practice outcomes (e.g. diagnosis, prescribing, referral practices) among healthcare providers, even when used alone [13].

In this study, we embraced the practice facilitation approach [17, 18] by engaging a mental health specialist to train and provide real time support supervision to the PHCPs in order to optimize results and to promote using the UCG to integrate mental health into routine practice [17, 18]. The participants liked the packaging of the summarized UCG on mental health conditions. They felt that the presence of the summarized guidelines eased their work, was time saving and was user friendly. They attributed their easy utilization of the guidelines to the presence of the summarized UCG and the training they received from the mental health specialist.

Our participants appreciated the training on mental conditions by the mental health specialist and expressed the need to provide ongoing training and support supervision. They appreciated the input and support of the mental health specialist and they valued the knowledge gained during training and support supervision. In this study, the mental health specialist regularly met the PHCPs at the respective intervention sites, following up on how they were fairing with using the intervention materials. Literature supports improved clinical care in those who receive on-going supervision [13]. During the supervising visits in this study, auditing of the register and the use of summarized UCG was done. Feedback was provided during the training aimed at optimizing the accuracy of data recorded. This result is consistent with other studies which found that interventions which include audit and feedback (alone or as a core component of a multifaceted intervention), compared with usual care, improved adherence to desired practice. Ivers et al. [31] suggested audit and feedback as a way of improving professional practice and healthcare outcomes [32]. In this study, participants acknowledged receiving mentorship to improve practice.

The participants appreciated that the modifications to the register to include the mental conditions improved the existing record in the HMIS registers. It did not require a new document to be made to be filled. They suggested adopting the summarized version of UCG which included mental disorders, and revision of the current HMIS registers to include recording mental health disorders. They further suggested advocating for the mental health agenda at the district level including increasing mental health awareness by easy to use educational tools for mental health such as charts or pictures that help in raising awareness among patients and the community about mental illness. Our participants preferred inclusion of the mental disorder in the HMIS register other than checklists. The office of the DHO came with its own recommendation of adding a checklist showing the full processes a clinician should follow when they get a patient. This addition did not work very well in this study because the PHCPs did not want to be reminded to do what they felt was now obvious (after the training). Our finding is consistent with other studies (Pantoja et al.) which remain uncertain on the value of checklists in improving adherence to guidelines [32].

Opinion leaders may be able to persuade healthcare providers to use the best available evidence when managing patients. These are individuals in a community or organisation who have a substantial influence on what the rest of the community or organisation does. In our study, we took the approach of engaging the office of the DHO Mbarara. The motive for engaging the local opinion leaders was to seek their input and invite them to officially introduce the intervention to the target population so as to have “political buy in” as there were many stakeholders in the field. Initially, we encountered resistance from various implementing partners at the HCs because there were other competing programs running at the study centers. After we engaged the office of the DHO to officially introduce the study, implementation became successful as there was improved healthcare workers’ adherence to desired practice. Indeed, our study participants suggested to have a district mental health focal person who could help push the mental health agenda.

In summary, our study attempted to build a systems policy intervention strategy to promote the use of the UCG in integrating mental health services into PHC. We employed a multifaceted approach of providing the summarized UCG (educational materials), modified registers to provide for information gathering, reminders, and support supervision and training to cater for knowledge sharing, as well as outreach visits, auditing of what is being done and feedback (monitoring and evaluation). Our findings were similar to the findings of researchers in the Cochrane reviews who found that combining the core policy intervention with other interventions led to a larger effect size than using the core intervention alone [13], which in our case was the summarized UCG. As an unintended outcome we found that some of the participants assumed over-confidence and enthusiasm in their ability to identify and manage all clients with mental conditions irrespective of the condition they were suffering from. These failed to adhere to the guidelines of referring to the next level as per the Ministry of Health [24] UCG.

### Limitations

The limitations in this study included the fact that the in-depth exploration of the feasibility and acceptability of the system policy intervention was conducted with a relatively modest sample of 7 interviews at 2 HCs (III and IV). These HCs were chosen for practical reasons as they were part of an initial study that identified the barriers and facilitators to integration of mental health services into PHC [11]. We purposively sampled PHCPs who had participated both in the initial study and the intervention because we were evaluating an intervention they contributed to. There is a need to rigorously evaluate whether our multifaceted intervention strategy to using the UCG improves uptake of integrating mental health services into PHC by repeating this study to larger scale and in totally different settings in Uganda.

### Implications from this study

The findings of this study may call for a rethinking of the referral guidelines and consideration of empowering PHCs at HC III level to treat and manage some of the common mental disorders they are expected to refer to HC IV. The same cadre of health professionals found at HC IV are also at HC III level although without a medical doctor. Secondly, there is a definite need to modify the HMIS registers to include the mental health problems to be recorded. Thirdly, when the Ministry of Health is supplying psychotropic medicines especially to HC III, there is need for an added guideline showing what the medicines are for (especially if they are meant for refills). Fourth, there are cost implications when it comes to scaling up the policy intervention to all HCs across the country. Although the cost of producing the summarized UCG and modifying the registers to include mental health were low, there will be need to carefully think through the training and supervision component at these lower HCs. The summarized guidelines alone and/or the addition to the register may not achieve the intended goal of promoting integration of mental health services into PHC.

## Conclusions and recommendations

Using a human cantered approach allowed us to develop a promising intervention to implement the Uganda Clinical Guidelines into PHC. Our study demonstrates that the use of the summarized UCG, modified HMIS registers to include mental health, training and support supervision by mental health specialists in implementing the UCG in integrating mental health at PHC settings is feasible and acceptable by the PHCPs in Mbarara district in south-western Uganda. Given the need for improved mental health care in Uganda, this intervention could be rigorously evaluated for effectiveness, scalability and generalizability.

## Additional files

**Additional file 1.** Description of the intervention.

**Additional file 2.** Summarized clinical guidelines.

**Additional file 3.** Checklist for clinician.

## Data Availability

Data on which this manuscript is based will not be publicly available since this work is still ongoing but will be available in future when the PhD program is completed.

## Abbreviations

DHO: District Health Officer
GUREC: Gulu University Research Ethics Committee
HC: health center
HMIS: Health Management Information System
OPD: Out-patients department
PHCP: primary health care providers
PHC: primary health care
Sida: Swedish International Development Cooperation Agency
SURE: Supporting the Use of Research Evidence
UMHCP: Uganda Minimum Health Care Package
UNCST: Uganda National Council for Science and Technology
WHO: World Health Organization
