# Supplementary material for: Implementing clinical guidelines to promote integration of mental health services in primary health care: a qualitative study of a systems policy intervention in Uganda
Source: Int J Ment Health Syst. 2019 Jul 17;13:49. doi: 10.1186/s13033-019-0304-9 (PMC6636121; doi:10.1186/s13033-019-0304-9)
Supplement: Supplementary file 1 — Additional file 1. Description of the intervention. [file 13033_2019_304_MOESM1_ESM.docx]

**Additional file 1: Description of the Intervention**

| **Level** | **Barriers** | **Intervention** | **Strategy** |
| --- | --- | --- | --- |
| Capability | Not comfortable/familiar using the guidelines | Training that focuses on mental health guideline recommendations | -Sensitize PCPs about the guidelines and how to use them |
|  |  | Making guidelines available | -Work with PCPs to choose content that should be put on the charts |
|  | limited knowledge about mental disorders | Support supervision to PCPs | -Provide real time support supervision to the PCPs |
|  |  | Training that focuses on skills in identification, management & referral of mental disorders | -Training workshop |
|  |  | Interactive learning / group training |  |
|  | Unfamiliar with what the guidelines say about mental health | Educational charts in examination rooms | -Pin charts in examination rooms |
|  | Insufficient time to keep referring to the guidelines |  |  |
| Opportunity | Guidelines unavailable or inaccessible | Easy access to guidelines | -Provide charts summarizing the guidelines on mental disorders |
|  | Not sensitized about the guidelines | Awareness campaign about the guidelines | -Provide sensitization meetings on presence and usage of the guidelines |
|  | No cues to remind them to use the guidelines | Educational charts in examination rooms | Pin charts in all examination rooms |
|  |  | Special registers to gather information on mental health | -Provide registers to collect mental health related information  -Regular review of registers |
| Motivation | Guidelines not practical | Adapt them to local setting | -Support supervision to the PCPs on guideline usage when screening for mental health |
|  | No specific measures to compel them to screen for mental health | Interest the District Health Officer to look out for mental health records | -Provide copies of report on mental health information collected to the office of the DHO |
|  | Fear of making a wrong diagnosis | Training on required skills | -Training workshop |
