# Supplementary material for: Implementing clinical guidelines to promote integration of mental health services in primary health care: a qualitative study of a systems policy intervention in Uganda
Source: Int J Ment Health Syst. 2019 Jul 17;13:49. doi: 10.1186/s13033-019-0304-9 (PMC6636121; doi:10.1186/s13033-019-0304-9)
Supplement: Supplementary file 2 — Additional file 2. Summarized clinical guidelines. [file 13033_2019_304_MOESM2_ESM.docx]

# **Additional file 2: Summarized clinical guidelines**

**MENTAL DISORDERS**

**1. Depression**

Is a common mental illness characterized by persistent sad or low mood, loss of interest in pleasurable activities (that the person used to enjoy), low energy, poor sleep, feelings of guilt, poor concentration, changes in weight or appetite, and suicidal wishes. If a person has had these signs and symptoms for over two weeks and has difficulty in day-to-day function do the following:

MANAGEMENT AND TREATMENT

HCIII

HCIV

Provide psychosocial support, if no response or severe (e.g Suicidal ideation), refer to HC IV

Consider antidepressant

**Amitriptyline**

- 50 mg at bedtime
- Increase by 25 mg every week aiming at 100-150 mg in divided doses or single bedtime dose by 4-6 weeks of treatment
- Useful in case of associated anxiety

*AVOID IN CHILDREN BELOW 12 YRS, ADOLESCENTS, ELDERLY, HEART DISEASES, SUICIDE* RISKS

**Fluoxetine**

- 20 mg once daily in the morning
- Start with 10 mg in the elderly
- Increase to 40 mg if not better after 4-6 weeks

*DO NOT USE IN CHILDREN BELOW 12 YEARS*

OR

Refer to District OR Regional Referral for further management

**2. Bipolar Disorder (Mania)**

Is a disorder of mood control characterized by episodes of an elevation of mood and increased energy and activity (mania) and in other occasions, there is a lowering of mood and decreased energy and activity (depression). If you see a patient with high/irritable mood, increased talkativeness, restlessness, over-activity OR decreased need for sleep, delusions, increased appetite but weight loss occurs due to over- activity and hallucinations do the following:

MANAGEMENT AND TREATMENT

HCIII

HCIV

**Carbamazepine**

- Initial dose 200 mg at night,
- Increase slowly to 600-1000 mg/day in divided doses

**Haloperidol**

- 5-10 mg every 12 hours then adjust according to response
- Up to 30-40 mg daily may be required in severe or resistant cases

**Chlorpromazine**

- Initially 100-200 mg every 8 hours, then adjust according to response
- Daily doses of up to 300 mg may be given as a single dose at night
- Gradually reduce the dose when symptoms resolve

ORA

Refer to District OR Regional Referral for further management

**NEUROLOGICAL**

**Epilepsy**

Is a chronic condition characterized by recurrent unprovoked seizures which are either convulsive OR non-convulsive.

**Convulsive epilepsy** a patient has sudden muscle contraction, causing them to fall and lie rigidly, followed by the muscles alternating between relaxation and rigidity with or without loss of bowel or bladder control

**Non-convulsive epilepsy** there may be change in awareness, behavior, emotions or senses (such as taste, smell, vision or hearing)

Consider a diagnosis of epilepsy if person has

- had at least 2 convulsive seizures in the last one year on two different days
- Seizures during an acute event (e.g. meningitis, acute traumatic brain injury) are not epilepsy.

**Clinical Features**

| **TYPE OF EPILEPSY** | **DESCRIPTION** |
| --- | --- |
| **Tonic Clonic** (grand-mal) | - May commence with a warning sensation in the form of sound, light or abdominal pain (aura) - There may be a sharp cry followed by loss of consciousness and falling - Tonic contraction (rigidity) of muscles occurs followed by jerking movements (clonic phase) - There may be incontinence of urine or feces, frothing, and tongue biting   A period of deep sleep follows |
| **Absence seizures** (petit mal) | - Mainly a disorder of children - A brief loss of consciousness (5-10 seconds) in which posture is retained but other activities cease - The child has a vacant stare - Previous activities are resumed at the end of the attack - Several attacks may occur in a single day |
| ***Status epilepticus*** | - A convulsive state in which the convulsions last less than 30 minutes - Several epileptic convulsions occur in succession without recovery of consciousness in between or convulsions not responsive to 2 doses of diazepam. - **THIS IS A MEDICAL EMERGENCY** |

**Tonic Clonic**

**Phenytoin**

- ***Adult*:** starting dose of 150-200 mg daily as single dose OR

2 divided doses and maintenance dose of 200-400 mg daily

- ***Child*:** starting dose of 3-4 mg/kg and maintenance dose of 3-8 mg/kg/day (max 300 mg daily)
- Increase slowly by 25-30 mg every 2 weeks

MANAGEMENT AND TREATMENT

HCIII

HCIV

**Phenobarbital**

- ***Adult*:** start dose 1mg/kg (60 mg) daily for 2weeks, if not controlled increase to 2 mg/kg (120 mg) for 2 months, if not controlled increase to 3 mg/kg (180 mg)
- ***Child*:** start dose 2 mg/kg/day for 2 weeks, if not controlled increase to 3 mg/kg for 2 months, if not controlled increase until maximum of 6 mg/kg/day

**Carbamazepine**

- ***Adult*:** start dose is 100-200 mg daily and increased in 100 mg increments every 1-2 weeks to a maintenance dose of 400 to 1400 mg daily
- ***Child*:** starting dose of 5 mg/kg/day and maintenance dose of 10-30 mg/kg/day in divided doses

*Given twice daily, steady state reached in 8 days*

OR

Convulsions not responsive to 2 doses of diazepam

**THIS IS A MEDICAL EMERGENCY**

Refer to District OR Regional Referral for proper diagnosis and management

OR

***Absence seizures and Status epilepticus***

***General principles***

- All suspected cases of non-convulsive epilepsy should be confirmed and treated by a specialist
- Convulsive epilepsy can be diagnosed and initially managed at hospital/HC4 level but drug refills should be available at lower level
- One brief isolated seizure does not need further treatment but review at 3 months and re-assess.

**SUBSTANCE USE DISORDERS**

**Alcohol use disorders**

Are conditions resulting from different patterns of alcohol consumption, including acute alcohol intoxication, harmful alcohol use, alcohol dependence syndrome and alcohol withdrawal state.

**Clinical features**

| **TYPE OF ALCOHOL DISORDER** | **DESCRIPTION** |
| --- | --- |
| ***Acute intoxication*** | - Transient condition following intake of alcohol resulting in disturbances of consciousness, cognition, perception, affect or behavior |
| ***Harmful alcohol use*** | - More than 5 drinks in any given occasion in the last 12 months - More than 2 drinks a day - Drinking every day |
| ***Alcohol dependence*** | - The need to take large daily amounts of alcohol for adequate functioning - The use of alcohol takes on a much higher priority for the individual than other behaviors that once had greater value |
| ***Alcohol withdrawal*** | - Tremor in hands, sweating, vomiting, hypertension, agitation, anxiety, headache, seizure and confusion in severe cases |

MANAGEMENT AND TREATMENT

HCIII

HCIV

**Alcohol dependence**

- Counseling and education of the patient
- Assess and manage concurrent medical and mental conditions
- Advise thiamine 100 mg daily

**Harmful alcohol consumption**

- Counseling and advice
- Investigate and treat concurrent medical or psychiatric illness (dementia, depression anxiety, psychosis etc.)

Refer to District OR Regional Referral for further management

**Alcohol dependence**

- Patient willing to stop

**NOTE: Alcohol consumption during pregnancy is extremely harmful for the baby: it can cause fetal alcohol syndrome. Counsel against any consumption**
