# Supplementary material for: Implementing clinical guidelines to promote integration of mental health services in primary health care: a qualitative study of a systems policy intervention in Uganda
Source: Int J Ment Health Syst. 2019 Jul 17;13:49. doi: 10.1186/s13033-019-0304-9 (PMC6636121; doi:10.1186/s13033-019-0304-9)
Supplement: Supplementary file 3 — Additional file 3. Checklist for clinician. [file 13033_2019_304_MOESM3_ESM.docx]

# **Additional file 3: Checklist for clinician**
